# Effects of carob rich-polyphenols on oxidative stress markers and physical performance in taekwondo athletes

**DOI:** 10.5114/biolsport.2022.106154

**Published:** 2024-06-17

**Authors:** Nawel Gaamouri, Hassane Zouhal, Katsuhiko Suzuki, Mehrez Hammami, Aloui Ghaith, El Hafedh El Mouhab, Anthony C. Hackney, Ismail Laher, Omar Ben Ounis

**Affiliations:** 1 Research Unit «Sport Performance, Health & Society», Higher Institute of Sport and Physical Education of Ksar Saîd, University of “La Manouba”, Tunis, Tunisia; 2 Higher Institute of Sport and Physical Education of Ksar Said, University of “La Manouba”, Tunis, Tunisia; 3 Univ Rennes, M2S (Laboratoire Mouvement, Sport, Santé) – EA 1274, F-35000 Rennes, France; 4 Faculty of Sport Sciences, Waseda University, Tokorozawa 359–1192, Japan; 5 Department of Exercise & Sport Science, University of North Carolina, Chapel Hill, NC, USA; 6 Department of Anesthesiology, Pharmacology and Therapeutics, Faculty of Medicine, University of British Columbia, Vancouver, Canada; 7 Tunisian Research Laboratory ‘‘Sport Performance Optimization’’, National Center of Medicine and Science in Sports, Tunis, Tunisia

**Keywords:** Polyphenols, Antioxidants, Ergogenic aids, Exercise

## Abstract

Excessive exercise can induce cell damage and impair muscle function by generating oxidative stress. Carob rich phenolic components have attracted the attention of many researchers because of their antioxidant actions. We utilized a double-blind randomized placebo-controlled design to study the putative antioxidant role of six weeks of daily polyphenol supplementation on selected blood markers of oxidative stress and performance in taekwondo athletes. We studied the effects of daily supplementation with carob (40 g/d, for six-weeks) on performance levels and antioxidant capacity in 22 taekwondo athletes (age 21.9 ± 1.2 years; height 1.66 ± 0.34 m; weight 68.3 ± 16.9 kg; women = 10, men = 12) using a randomized, double-blinded study. Participants were divided into an experimental group (EG) or placebo group (PG). All athletes performed a frequency speed of kick test mult (FSKT_mult_) before and after a six-week training period. Superoxide dismutase (SOD), catalase (CAT), and malondialdehyde (MDA) were measured 5 min after a FSKT_mult_. Physical performances improved significantly after six weeks in EG compared to PG for kicks number per set (from set 1 to set 5: p = 0.032, d = 0.70; p = 0.020, d = 0.77; p = 0.001, d = 1.12; p = 0.001, d = 1.25; p = 0.003, d = 1.01), total kicks number (p = 0,002, d = 1.04), and kick decrement index (%) (p = 0.017, d = 0.13). There were significant increases in CAT (p = 0.001, d = 1.85) and SOD (p = 0.001, d = 1.98) activities and significant decreases in MDA levels (p = 0.002, d = 0.81) in the EG. Carob supplementation during a six-week training program reduced oxidative stress and improved physical performance levels in taekwondo athletes.

## INTRODUCTION

Excessive and poorly monitored training (including aerobic [1] and anaerobic [2] exercises), as sometimes practiced by athletes, often leads to increases in oxidative stress [3]. Sports involving combat maneuvers frequently contain intense physical activities that are dependent on both aerobic and anaerobic energy metabolism [4]. Competitions such as taekwondo involve high levels of physiological, hormonal and psychological stress [5–7]. Taekwondo competitions require athletes to be physically fit, where power is generated through aerobic and anaerobic metabolism, muscular strength, speed and agility [8]. All fights in taekwondo competitions (from qualification to finals) occur in a single day [9]. The frequency speed of kick test (FSKT10s) is the only assessment that is mainly related to the ATP-PCr energy system, due to its intense nature and short duration (10s), and which is performed intermittently (5 × 10s/10s intervals, FSKT_mult_) [10]. Moreover, this test can be easily applied in the taekwondo training facility and does not require sophisticated equipment [11–13]. Additionally, the FSKT is sensitive enough to identify acute [14] and long-term performance changes [13], and is able to discriminate between international/ national and state/regional female taekwondo performances [11]. Engaging in taekwondo necessitates periods of high-intensity efforts, leading to cellular generation of reactive oxygen species (ROS) [2], oxidative stress and cellular damage [15, 16]. Accumulation of ROS induces cell and skeletal muscle damage, and causes rapid musclefatigue and reduces exercise performance [17]. Therapeutic interventions including massage, cryotherapy and nutritional strategies have been studied in efforts to reduce oxidative stress and improve athletic performance [18, 19]. A recent review summarized the findings of studies on the effects of natural products (e.g., vitamin C, vitamin E, β-carotene, quercetin, resveratrol, and polyphenols) either alone or in combination with other supplements on exercise-induced oxidative stress [20].

Polyphenols are natural compounds that can prevent or reduce oxidative stress, relieve muscle soreness and improve performance [21, 22, 23]. To this end, carob is rich in dietary fiber, polyphenols, and cyclitols with low amounts of fat and is gaining increasing use due to its ability to improve health and prevent numerous chronic diseases [24]. Carob has antiproliferative and apoptotic activity against cancer cells, can treat diarrhea symptoms, and possesses antihyperlipidemic and antidiabetic effects [25]. Furthermore, carob fiber has a higher antioxidant capacity than many other foods rich in polyphenols such as blueberries, grapes or red wine [26]. Only a few studies have investigated the effects of carob supplementation on physiological stress in athletes undertaking aerobic and anaerobic activities. We utilized a double-blind randomized placebocontrolled design to study the putative antioxidant role of six weeks of a daily polyphenol supplementation with carob on selected blood markers of oxidative stress [malondialdehyde (MDA), superoxide dismutase (SOD) and catalase (CAT)] and performance in taekwondo athletes. We hypothesized that carob supplementation reduced oxidative stress and improved physical performance levels in taekwondo athletes.

## MATERIALS AND METHODS

### Participants

Twenty-two taekwondo athletes (age: 22.7 ± 2.4 years, see Table 1) who are students at the High Institute of Sport and Physical Education of Ksar Said (Tunis, Tunisia) volunteered to participate in the current study. Participants were recruited following a poster submitted to the Institute’s Sports Department. The poster included a brief description of the study, its objectives, the inclusion and exclusion criteria and the tasks required from participants. All participants were required to be at the level of black belts (first Dan or greater).

**TABLE 1:** Characteristics of athletes.

Measures	Groups	T1	T2	P
**Weight (kg)**	**PG**	72.9 ± 15.2	70.3 ± 13.3	
	**EG**	70.8 ± 12.6	68.8 ± 12.4	> 0.05

**Height (m)**	**PG**	1.72 ± 0.19	1.72 ± 0.15	
	**EG**	1.72 ± 0.11	1.72 ± 0.07	> 0.05

**BMI (kg/m^2^)**	**PG**	24.5 ± 4.9	23.7 ± 4.3	
	**EG**	23.9 ± 3.8	23.2 ± 3.7	> 0.05

Values are presented as mean ± SD. Variables were measured before (T1) and after (T2) six-weeks of carob supplementation. The data were analyzed using two-way ANOVA: two groups (EG, PG) × two time points (T1, T2). EG: experimental group, PG: placebo group.

Inclusion criteria of participants were: i) they trained at least three times a week for 90 to 120 min per session, ii) if they participated at a national level for at least three years, iii) if they were not consuming antioxidants or anti-inflammatory compounds, either currently or one month prior to the study, iv) females should not be consuming any form of oral contraception and physical performance was measured during the luteal phase of the menstrual cycle. Participants were excluded if they were smokers, sustained an injury in the previous 30 days, had recent surgery, had hypertension or cardiovascular/ metabolic disorders. The study procedures were explained before participants provided written informed consent, and participants were informed that they could withdraw from the study at any stage. The study was approved by the Ethical Committee on Human Research of the University of la Manouba, Tunisia (Approval Number: 20170396), and was carried out in accordance with the Declaration of Helsinki. The study lasted six-weeks, and during this period, athletes trained four times per week with each session lasting 90 minutes. Each training session included specific technical interval training (IT), tactical and technical training sessions (TT), technical-development training sessions and free taekwondo sparring, and was supervised by the same taekwondo coach throughout the study. The training sessions included specific technical interval training, tactical technical training sessions, technical-development training sessions and free taekwondo sparring [8].

### Experimental design

We used a double-blind randomized design to study whether supplementation with carob for 6 weeks improved antioxidant capacity and physical performance of taekwondo athletes. Athletes were randomly assigned to one of two study groups: A) Supplement Group (EG: n = 11; 5 females and 6 males) and B) Placebo Group (PG: n = 11; 5 females and 6 males).

**FIG. 1:**
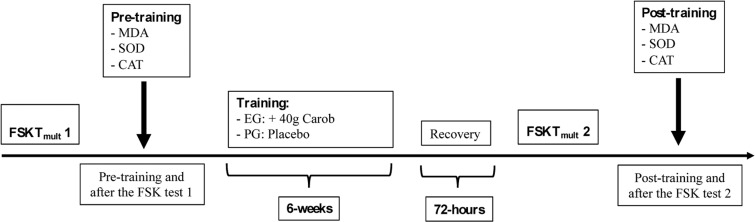
Experimental design FSK: frequency speed of kick; EG: experimental group; PG: placebo group

Participants successfully completed two familiarization trials over a two-week period before the data were collected immediately before the training period (pre- and post-exercise) and after the six-week trial (pre- and post-exercise) (Figure 1). Testing involved the frequency speed of kick test mult (FSKT_mult_) protocol; the athletes all followed identical training sessions and data were recorded at the same of every day of the tests. A daily record of nutrient intake was collected from each participant seven days before starting the study; the participants were instructed in detail (written and oral explanations) on these data collection procedures. All participants continued with their usual eating habits and were instructed to be accurate with recording the amount and type of food and fluid intake. Common measures (e.g., cups and tablespoons) and quantities (e.g., grams) were provided to the participants, and individual diets were calculated using the Bilnut 4 software package (SCDA Nutrisoft, Cerelles, France) and food composition tables published by the Tunisian National Institute of Statistics in 1978. Nutrient intakes were within the reference dietary guidelines for healthy Tunisian adults.

### Exercise protocol

Frequency Speed of Kick Test Mult (FSKT_mult_): The Frequency Speed of Kick Test (FSKT_10s_) lasts for 10 s, and during its execution each athlete is placed in front of a stand bag equipped with a simple trunk taekwondo protector. After a command, the athlete performs the maximal number of kicks possible, alternating the right and the left legs. The turning kick, known as Bandal Tchagui, is used during the test [12, 14]. The same procedures was used in the FSKT_10s_ were also used during the FSKT_mult_. Briefly, five FSKTs with a 10-s rest interval between repetitions were executed. Performance was determined by the total number of kicks in each set, the total number of kicks in five sets, and the kick fatigue index [10] [12]. The FSKT_mult_ had a mean intraclass correlation coefficient (ICC) of 0.85 between tests and retests. The mean test and retest coefficient of variation was 3.9%. The kick decrement indicates performance decreases during the tests.

The number of kicks applied during the multiple FSKT is considered when calculating the kick decrement. The equation takes into account the results of all FSKT sets [27]. Kick decrement index (%) = [1 – (FSK test 1 + FSK test 2 + FSK test 3 + FSK test 4 + FSK test 5 / best FSK test × number of sets)] × 100.

### Sample collection

Blood samples were always collected at the same time (7 to 8 AM) following an overnight (12 hr) fast. Venous blood samples were collected by the same technician at the following times: A) pre-training, after the FSKT_mult_ (Pre); and B) post-training program, after the FSKT_mult_ (Post). Blood samples were taken from the antecubital site and placed in EDTA tubes (Vacutainer, Becton Dickinson, Franklin Lakes, NJ, USA) after the participants were seated for 5 min immediately after the test. Blood samples were transported to the laboratory in crushed ice and then centrifuged (3500 × g at 4° C for 12 min) and stored in aliquots (-80°C) for later analysis. Inter-assay variance was minimized by analyzing samples in the same assay run; measurements were made in duplicate using an automated analyzer (Beckman Coulter UniCel® DxC 600 Synchron®, Beckman Instruments, California, USA), using reagents, standards, and controls from Randox Laboratories (Crumlin, Northern Ireland, UK). The measurements had an intra-assay coefficient of variation of less than 7%.

### Oxidative stress and antioxidant enzymes

Plasma lipid peroxidation was estimated by measuring malondialdehyde (MDA) levels using a double heating method [28]. Aliquots (250 μl) from the sample were mixed with butylated hydroxytoluene –trichloroacetic acid (TCA) Q3 solution (250 μl) containing 1% BHT (m/v) dissolved in 20% TCA (m/v) and centrifuged at 1,000 × g for 5 min at 4 °C. The supernatant (400 μl) was mixed with 0.5 N HCl (80 μl) and 120 mM thiobarbituric acid (TBA) in 26 mM Tris (320 μl) and then heated (80° C for 10 min). The absorbance of the resulting pink chromophore was measured at 532 nm using a SmartSpec 3000 Bio-Rad UV-visible spectrophotometer (Germany).

Concentrations of MDA were calculated (using an extinction coefficient of 155 mM^-1^cm^-1^ for the MDA -TRA complex) using the formula: MDA concentration (M) = absorbance at 532 nm/1.56 × 105. Superoxide dismutase (SOD) activity was determined by the epinephrine method [29], which is based on the capacity of the SOD to inhibit the auto-oxidization of the epinephrine in adrenochrome while trapping the flux of anion superoxide. The sample was mixed in a buffer of Na_2_CO_3_/NaHCO_3_ (50 mM, pH = 10.2) with 10 μl of bovine CAT (0.4 U/ml) and 20 μl of epinephrine (5 mg/ml). The SOD activity was assessed by spectrophotometry. Detection was performed at 480 nm with a Bio-Rad spectrophotometer (Bio-Rad Laboratories, Philadelphia, USA).

CAT is a metalloenzyme that transforms the peroxide of hydrogen in water and molecular oxygen according to the following reaction: 2H_2_O_2_ → 2H_2_O + O_2_. The sample was mixed with phosphate buffer (50 mM, pH = 7). The kinetics of the CAT activity was spectrophotometrically measured at 240 nm for 3 min while measuring the decomposition of the H_2_O_2_. The CAT activity was calculated according to the Beer–Lambert principle.

### Determination of total phenols Extraction

A test sample of degreased vegetable material (2 g) was macerated in a mixture of acetone / water (100ml, 70/30 v/v) for 24 hours at room temperature before being vacuum filtered. The acetone / water mixture was then evaporated (45° C) to dryness under reduced pressure, and the resultant residue recovered with 3 ml methanol for later assays [30].

### Dosage by Folin-Ciocalteu method

The process requires the reduction of phoshomolybdic acid in FolinCiocalteau reagent (a yellow acid consisting of acid polyheterocycles containing molybdenum and tungsten) by polyphenols under alkaline conditions [31]. This reaction creates a dark blue tungsten molybdenum complex, which is measured using a spectrophotometer.

### Procedure

Polyphenols were measured using the method of Singleton as reported by Dogyan et al. [32]. The residue resulting from the extraction was dissolved in distilled water (5 ml), and 100 μl of this solution was diluted to 3 ml and added to 0.5 ml of Folin Ciocalteu reagent. This reaction was allowed to proceed for 3 min, after which 2 ml of 20% a sodium carbonate solution was added; the mixture was vortexed and incubated in the dark for 1 hr, and the absorbance was read at 650 nm.

### Carob extract and placebo supplementation

Mature carob pods collected from the north-west of Tunisia and dried in an incubator (50° C, 72 h) and then powdered in an electric blender (Moulinex Ovatio 2, France) to yield a mixture of carob pulp (90%) and carob seeds (10%). Each 40 g sample of carob powder contained 208 mg of total polyphenol, and 14.4 mg of flavonoids. Participants in both groups were contacted by telephone to remind them to consume the supplements at the agreed times. The placebo was prepared with a carob-flavored commercial drink (250 ml) containing water, citric acid, natural flavor (carob), sweeteners (aspartame × 0.3 g/l), acesulfame K (0.16 g/l)), stabilizers (Arabic gum) and was free of antioxidants, vitamins and polyphenols. Participants in the EG consumed freshly prepared carob powder (40 g) diluted in 250 ml of water daily at breakfast for 6 weeks.

### Statistical analysis

Data are reported as mean ± standard deviation (SD) and normality was verified with the Kolmogorov-Smirnov test. A two-way ANOVA (2 levels [supplementation (PG and EG) × 2 levels [training period (Pre and Post)] was used to determine the effects of carob supplementation on physical performance (FSK test) and biochemical measurements. No between sexes comparison were conducted due to the small sample numbers for men and women. The Cohen’s d test was used to assess effect size (ES) under the two conditions, so that a d value of 1 indicates that the two groups differed by 1 SD.

ES were considered to be either trivial (d < 0.2), small (d = 0.2–0.6), moderate (d = 0.6–1.2), large (d = 1.2–2.0) and very large (d = 2.0–4.0) [33]. Relationships between parameters were assessed using the Pearson’s product-moment correlation coefficient (r), which were interpreted as trivial (r < 0.1), small (0.1 < r < 0.3), moderate (0.3 < r < 0.5), large (0.5 < r < 0.7), very large (0.7 < r < 0.9), nearly perfect (0.9 < r < 1), and perfect (r = 1) [34]. Statistical analyses were made with a SPSS (SPSS Inc., Chicago, IL, version 20.0), and significance was set at p < 0.05.

## RESULTS

There were no significant differences in the study groups for any of the anthropometric characteristics (Table 1). There were significant weight reductions when comparing pre- and post-interventions (~2 kg) in both PG and EG, but these were not different between the groups.

### Effect of carob supplementation on oxidative stress and antioxidant enzymes

Blood levels of oxidative stress and antioxidant enzymes are shown in Table 2. MDA levels following the FSKT_mult_ decreased after the supplementation period for both EG (p < 0.01) and PG (p = 0.01). The decrease was significantly higher in EG (-25.6 ± 6.1%). In addition, SOD levels increased after the supplementation period compared to values before supplementation for EG (p < 0.01) and PG (p < 0.01). Values were significantly higher in EG (+32.7 ± 5.1%). Similarly, values of CAT increased after the supplementation period for EG (p < 0.01) and PG (p < 0.01). Values were significantly higher in EG (+21.0 ± 0.4%). There were significant differences (using ANOVA) for group × time interactions for SOD activity (p = 0.001, d = 1.85), CAT activity (p = 0.001, d = 1.98) and MDA levels (p = 0.002, d = 0.81).

**TABLE 2:** Changes in blood levels of oxidative stress markers induced by the FSKT_mult_ before and after 6-week supplementation in both groups (PG and EG).

Measures	Groups	Pre-Supplementation	Post-Supplementation	MΔ ± SDΔ (%)	Cohen’s d	ANOVA p-value (Cohen’s d)		
						Time	Group	Time × Group
**MDA (nmol/ml)**	**EG**	0.35 ± 0.01	0.26 ± 0.01^**^	-25.6 ± 6.1	-6.42	0.001	0.001	0.002
	**PG**	0.35 ± 0.01	0.30 ± 0.01^**^	-16.1 ± 4.5	-4.75	(3.64)	(1.33)	(0.81)

**SOD (KL/ml)**	**EG**	0.34 ± 0.01	0.46 ± 0.02^**^	32.7 ± 5.1	7.77	0.001	0.001	0.001
	**PG**	0.34 ± 0.01	0.40 ± 0.01^**^	17.0 ± 4.6	4.40	(5.69)	(1.36)	(1.85)

**CAT (Mm/ml)**	**EG**	18.20 ± 0.25	22.04 ± 0.30^**^	21.0 ± 0.4	15.41	0.001	0.001	0.001
	**PG**	18.24 ± 0.27	21.17 ± 0.30^**^	16.1 ± 1.9	11.06	(15.78)	(0.85)	(1.98)

Values are presented as mean ± SD. Blood samples were collected after the FSKTmult, before the six-week treatment (Pre-supplementation) and after the treatment (Post-supplementation). Data were analyzed using two-way ANOVA: two groups (PG and EG) × two time points (Pre, Post). EG: experimental group, PG: placebo group; Superoxide dismutase: SOD; catalase: CAT; malondialdehyde: MDA; EG: experimental group; PG: placebo group; MΔ ± SDΔ: percentage change of blood parameters ± SD;

** : p < 0.05.

### Effect of carob supplementation on FSKT_mult_ performance

Physical performance measured during the FSKT_mult_ (i.e., set 1 to set 5, total number and F-index) after PLA and carob supplementation are shown in Table 3.

**TABLE 3:** Values (mean ± SD) of FSKT_mult_ parameters before (T1) and after (T2) supplementation.

Measures	Groups	T1	T2	ANOVA	
				Time × Group	Cohen’s d
**Set 1**	**PG**	21.63 ± 1.12	22.18 ± 1.16	p = 0.032	0.70
	**EG**	21.81 ± 1.77	24.18 ± 1.25		

**Set 2**	**PG**	20.72 ± 1.19	21.18 ± 1.16	p = 0.020	0.76
	**EG**	21.27 ± 1.84	23.81 ± 1.40		

**Set 3**	**PG**	19.90 ± 0.83	20.18 ± 0.98	p = 0.001	1.12
	**EG**	20.00 ± 1.73	23.09 ± 1.51		

**Set 4**	**PG**	18.90 ± 0.94	19.54 ± 0.93	p = 0.003	1.24
	**EG**	19.09 ± 1.70	22.90 ± 1.57		

**Set 5**	**PG**	18.45 ± 1.29	19.18 ± 1.07	p = 0.002	1.01
	**EG**	18.63 ± 1.96	22.27 ± 1.55		

**Total Number**	**PG**	99.63 ± 4.58	102.27 ± 5.00	p = 0.002	1.04
	**EG**	100.22 ± 6.70	109.27 ± 9.26		

**KDI**	**PG**	8.18 ± 2.40	7.27 ± 2.72	p = 0.017	0.79
	**EG**	8.81 ± 2.67	4.27 ± 1.73		

EG: experimental group, PG: placebo group; T1: before supplementation period; T2: after supplementation period, KDI: Kick decrement index.

An ANOVA test revealed significant group × time effects for all parameters tested: set 1 (p = 0.032, d = 0.70), set 2 (p = 0.020, d = 0.77), set 3 (p = 0.001, d = 1.12), set 4 (p = 0.001, d = 0.12), set 5 (p = 0.003, d = 1.01), and the total number (p = 0.002, d = 1.04).

Similarly, an ANOVA test revealed significant group × time effects for the kick decrement index (p = 0.017, d = 0.13). The relationship between percentage changes of antioxidant enzymes and performance markers during the FSKT_mult_ is shown in Table 4. Correlations between % changes of SOD and FSKT_mult_ parameters (best, total number and kick decrement index, respectively) were either positively moderate, Kick decrement index. positively very large and negatively large between % changes of SOD and FSKT_mult_ parameters (best, total number and kick decrement index, respectively). There were negative small correlations, negative moderate correlations and positive very large correlations between % changes of MDA and FSKT_mult_ parameters (best, total number and kick decrement index, respectively).

**TABLE 4:** Relations between percentage changes of blood parameters of antioxidant enzymes and those of performance markers after the FSKT_mult_ both before and after the training program.

		% changes of blood parameters of oxidative stress		
		SOD	CAT	MDA
**Performance parameters changes during FSKT_mult_ (%)**	**Best**	r = 0.48^**^ (moderate)	r = 0.52^**^ (large)	r = - 0.21^**^ (small)
	**Total Number**	r = 0.72^**^ (very large)	r = 0.80^**^ (very large)	r = - 0.46^**^ (moderate)
	**KDI**	r = - 0.63^**^ (large)	r = - 0.72^**^ (very large)	r = 0.72^**^ (very large)

** : p < 0.05. Superoxide dismutase: SOD; catalase: CAT; Malondialdehyde: MDA; EG: experimental group; PG: placebo group KDI: Kick decrement index.

## DISCUSSION

The results of our study indicate that performance in a six-week taekwondo training program was improved by carob supplementation in both treatment (EG) and placebo (PG) groups. Our finding that carob improved aerobic performance in taekwondo athletes supports previous studies reporting improved physiological effects of polyphenols on athletes. Polyphenols reportedly augment vasodilation by increasing endothelial nitric oxide synthesis [35, 36], which improves tissue perfusion of both oxygen and other substrates to active muscles during exercise, and so enhances exercise performance [37, 38]. The low levels of fatigue index shown in the EG reinforced the notion that the direct effects of polyphenols may involve the reduction of muscle fatigue at the level of contractile function [39]. Thus, according to Kashi et al. [40], the ergogenic effects of fruit-derived polyphenols may be associated with improved vascular function, enhanced muscle perfusion and enhance oxygen extraction, and improved physical work capacity.

Our study indicates that carob supplementation improved antioxidant status in athletes, likely due to augmented activities of SOD and CAT. Furthermore, six weeks of daily supplementation with carob significantly decreased MDA levels and enhanced physical performance. Acute consumption of cocoa rich polyphenol reduces oxidative stress and muscle damage induced by exercise [41], while chronic intake of dark chocolate reduces oxidative stress and muscle damage biomarkers in elite football players undergoing intensive physical training [26, 42]. The beneficial effects of dark chocolate support the hypothesis that polyphenols, particularly epicatechin, may be responsible for this effect [42]. The ability of carob to increase antioxidant (SOD and CAT) activities may be related to inhibition of NADPH-oxidase activity and reduced superoxide anion overproduction [43].

Our study also examined the effects of carob supplementation on antioxidant parameters in response to intense exercise. The % change of performance was correlated with the % change of antioxidant enzyme (SOD and CAT) and MDA levels for the most part. Some authors describe the antioxidant capacity conferred by supplementation with fruit-derived polyphenols as being ergogenic [40]. In addition, aqueous extracts of carob protect cells from lipid peroxidation induced damage. Our results are in agreement with the findings of Al-Olayan et al. [44] who suggested that carob prevents liver glutathione depletion and stabilizes membrane permeability by inhibiting MDA formation and reducing lipid peroxidation in mice.

The efficacy of the carob preparation may be related to its high content of polyphenols, which have antioxidant effects [45]. Other studies reported that supplementation with such antioxidants accelerates recovery from physical fatigue [46] and prevents exerciseinduced tissue damage by limiting exercise-induced muscular and oxidative damage, inflammation, and the consequences of reduced muscle force capacity [47]. Carob supplementation also reduce tissue oedema and/or the accumulation of metabolic by-products [48].

### Limitations and Implications for future Research

Taekwondo is an intermittent combat sport and competition rules require that athletes recover quickly between fights, and all contests (from qualification to finals) occur in a single day. To our knowledge, this is the first study to evaluate carob supplementation on antioxidant capacities and physical performance in taekwondo athletes. However, our study has a number of limitations that should be addressed. First is the relatively small sample size of our study groups and the limited number of measurement time points. Hence studies with larger sample sizes are needed to verify these findings. Secondly, we measured only three markers of oxidative stress; measuring other biomarkers of antioxidant activity such as Ferric-reducing ability of plasma (FRAP), total antioxidant status (TAS), 2.2’-azino-bis-3-ethylbenzthiazoline-6-sulphonic acid (ABTS) would have added to our findings. Lastly, we did not measure nitric oxide biomarkers (e.g., plasma nitrate, nitrite and L-citrulline levels) which could have validated the results obtained in performance tests [51]. Finally, we did not address the mechanistic basis of the biological benefits of supplementation with carob, for example by measuring skeletal muscle eNOS activity [51]. Hence, a causal role for the benefits of carob use can only be claimed after additional studies are performed.

### Practical Applications

The decrease of oxidative stress which improved physical performance should interest taekwondo coaches, as it is relatively easy to incorporate, it is easily possible to incorporate 40 g of carob in the diet of an athlete for at least six-weeks before a competition.

## CONCLUSIONS

The intense muscle-endurance tests used in this study induced oxidative stress, muscle damage and fatigue in taekwondo athletes. The results of our study indicate that daily supplementation with 40 g of carob-rich polyphenol for six-weeks prevents oxidative stress as induced by high-intensity exercise and improves physical performance in taekwondo athletes.
